# Genetic testing in ALS: A survey of current practices

**DOI:** 10.1212/WNL.0000000000003686

**Published:** 2017-03-07

**Authors:** Alice Vajda, Russell L. McLaughlin, Mark Heverin, Owen Thorpe, Sharon Abrahams, Ammar Al-Chalabi, Orla Hardiman

**Affiliations:** From the Academic Unit of Neurology (A.V., R.L.M., M.H., O.T., O.H.), Trinity College Dublin; Cognitive Aging and Epidemiology Centre (S.A.), University of Edinburgh; and Institute of Psychiatry, Psychology and Neuroscience (A.A.-C.), King's College London, UK.

## Abstract

**Objective:**

To determine the degree of consensus among clinicians on the clinical use of genetic testing in amyotrophic lateral sclerosis (ALS) and the factors that determine decision-making.

**Methods:**

ALS researchers worldwide were invited to participate in a detailed online survey to determine their attitudes and practices relating to genetic testing.

**Results:**

Responses from 167 clinicians from 21 different countries were analyzed. The majority of respondents (73.3%) do not consider that there is a consensus definition of familial ALS (FALS). Fifty-seven percent consider a family history of frontotemporal dementia and 48.5% the presence of a known ALS genetic mutation as sufficient for a diagnosis of FALS. Most respondents (90.2%) offer genetic testing to patients they define as having FALS and 49.4% to patients with sporadic ALS. Four main genes (*SOD1*, *C9orf72*, *TARDBP*, and *FUS*) are commonly tested. A total of 55.2% of respondents would seek genetic testing if they had personally received a diagnosis of ALS. Forty-two percent never offer presymptomatic testing to family members of patients with FALS. Responses varied between ALS specialists and nonspecialists and based on the number of new patients seen per year.

**Conclusions:**

There is a lack of consensus among clinicians as to the definition of FALS. Substantial variation exists in attitude and practices related to genetic testing of patients and presymptomatic testing of their relatives across geographic regions and between experienced specialists in ALS and nonspecialists.

Amyotrophic lateral sclerosis (ALS) is categorized into familial (FALS) and sporadic (SALS) forms. However, the recognition of FALS is limited by incomplete penetrance of disease-causing mutations and variable family size, which can result in apparently sporadic presentation of familial disease.^1,2^ Heritability estimates for SALS are high,^3–5^ relatives of patients with SALS are at increased risk,^6^ and mutations known to cause FALS have frequently been observed in apparently sporadic cases of ALS.^7^ Familial forms of the condition are often characterized by reduced penetrance and genetic pleiotropy, and there is evidence of both oligogenic and polygenic inheritance in apparently sporadic disease.^8^ In addition, ancestral origin is important, with variation in the frequency of ALS genes in different patient populations.^7,9–11^

Increasing knowledge of the genetic architecture of ALS suggests that the majority of future discoveries are likely to involve a multitude of rare, de novo, or low-effect risk variants and disease-causing mutations.^12^ Given these complexities, decisions by clinicians as to whom to refer for genetic testing and which genes to test require detailed knowledge of a rapidly changing genetic landscape. Published consensus guidelines from 2012 have quickly been superseded by new findings^13^ and formal provision of genetic testing for those who have a first- or second-degree relative with ALS or frontotemporal dementia (FTD) is now recommended,^14^ as is a consideration for genetic testing of apparently sporadic disease, and recognition that each ancestral population will have a different genetic profile. Guidelines for presymptomatic testing in a research setting that includes predecision, pretest, and posttest counseling have also been published.^15^

Using these existing recommendations, we have evaluated the international consensus on the use and perceived value of genetic testing in ALS, and have assessed the factors that determine the extent to which ALS clinicians recommend genetic testing in a clinical setting.

## METHODS

A questionnaire on attitudes toward genetic testing for ALS was designed following consultation with neurologists specializing in ALS (supplemental data at Neurology.org). Participants were asked to respond to questions about their opinions of the definition of FALS, their use of genetic testing in clinical practice for patients and presymptomatic family members, and their own personal preference for testing if they were a patient with ALS. The known ALS genes included in the survey were *SOD1*, *C9orf72*, *TARDBP*, *FUS*, *ANG*, *VABP*, *SETX*, *DCTN1*, *VCP*, *UBQLN2*, *PFN1*, and *ATXN2.* These were selected as being the most likely to be tested based on their importance and their association with specific ancestral populations and clinical phenotype. Demographics information was collected to allow comparison of the opinions of clinicians from different countries and with different levels of experience. Twenty-one questions were uploaded to Survey Monkey (surveymonkey.com) and the link was circulated by members of ALS networks from Europe (the European Network for the Cure of ALS), the United States (the Northeast ALS Consortium), Canada (the Canadian ALS Research Network), Australia, Japan, and South America. Statistical analyses were performed using R version 3.0.2 using Fisher exact test or linear regression.

## RESULTS

### Demographics.

A total of 167 clinicians (116 male, 51 female) from 21 countries responded to the survey. Respondents were mainly neurologists (86.8%) and trainee neurologists (10.8%). Most were from academic university hospitals (85.6%), with the remainder working at general hospitals (9.6%) or private hospitals and clinics (4.8%). A total of 50.9% of those questioned see more than 30 new patients with ALS each year, 9.6% see 21–30, and the remainder (39.5%) see fewer than 20. The majority stated that they have a special interest in ALS (83.2%) and of those 23.0% had been specializing in the field for more than 20 years, 34.5% for 10–20 years, and 42.5% for less than 10 years.

### Definition of FALS.

**Figure 1:**
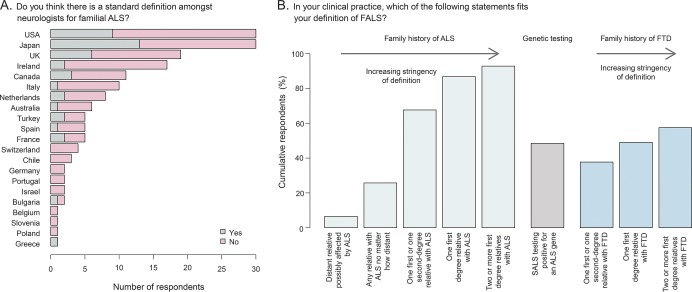
Definition of familial amyotrophic lateral sclerosis (FALS) (A) Country-specific variation in opinion on the existence of a standard definition for FALS. (B) FALS definitions used by respondents. Within amyotrophic lateral sclerosis (ALS) or frontotemporal dementia (FTD) disease categories, choice of a lower-stringency definition automatically meant higher stringency definitions were also selected. SALS = sporadic amyotrophic lateral sclerosis.

The majority of those surveyed (73.3%) did not consider that there is a standard definition among neurologists for FALS (figure 1A). This proportion did not differ significantly when considering broad geographic regions (the Americas, Europe, and Asia-Pacific; *p* = 0.17, Fisher exact test) or individual countries (*p* = 0.66).

Respondents were provided with a list of FALS definitions and asked to select all those that fitted their definition in clinical practice (figure 1B). As the stringency of the FALS definition increased (i.e., as the family members with ALS became more closely related and increased in number), so did the number of respondents who accepted that definition. A total of 48.5% of respondents considered testing positive for a known ALS gene to be sufficient to meet the criteria for FALS, and the presence of a family history of FTD was considered relevant by 57% of respondents. Respondents who identified themselves as having a special interest in ALS were more likely to use less strict definitions of FALS, and for example, include patients with 1 first- or 1 second-degree relative with ALS in their definition (73.38% vs 39.29%, *p* = 7.7 × 10^−4^, Fisher exact test). Similarly, 42% of those with a special interest, but only 14% of nonspecialists, agreed that the presence of a first- or second-degree relative with FTD met the criteria for FALS (*p* = 5.1 × 10^−3^, Fisher exact test). Those who see more than 30 new patients with ALS per year were significantly more likely than those who see fewer to define this case as FALS (52.9% vs 22.0% [*p* = 5.6 × 10^−5^]). Fifty-two percent of specialists use detection of a known gene in a sporadic case as one of their definitions of FALS, compared to 28.57% of nonspecialists (*p* = 2 × 10^−2^, Fisher exact test). Those with more than 15 years' experience and those who see more than 30 new patients a year were more likely to include this in their definition of FALS compared to those who have less experience (*p* = 6.1 × 10^−3^ and *p* = 0.02, respectively).

### Use of genetic testing in clinical practice.

**Figure 2:**
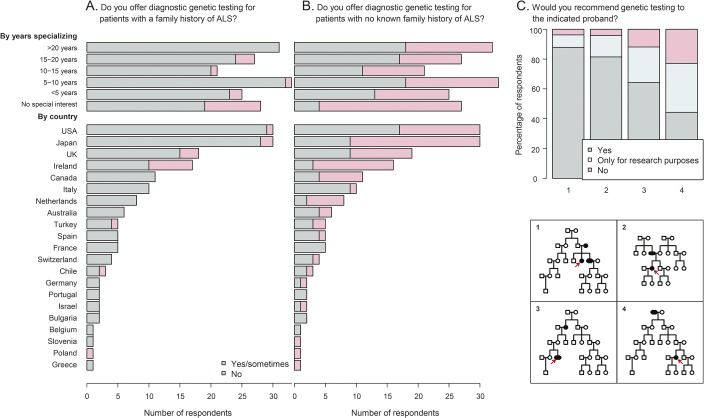
Genetic testing of patients with and without a family history of amyotrophic lateral sclerosis (ALS) (A) Diagnostic genetic testing offered to patients with a family history of ALS (yes/no). (B) Diagnostic genetic testing offered to patients with no known family history of ALS (yes/no). (C) Genetic testing recommended to indicated proband (yes/no).

The majority of respondents (90.2%) stated that they offer diagnostic testing for patients who meet their definition of FALS, while 49.4% of respondents stated that they offer genetic testing to patients with no known family history of ALS (figure 2, A and B). Those with a special interest in ALS were significantly more likely to offer genetic testing, both in cases of FALS (*p* = 1.8 × 10^−4^) and SALS (*p* = 9.1 × 10^−5^). Experienced ALS specialists (those specializing in ALS for more than 15 years) were not significantly different from less experienced specialists in their decision to test patients with positive or negative family histories of ALS (*p* = 1.0 and 0.49, respectively, Fisher exact test). However, respondents who see more than 30 new patients with ALS per year were significantly more likely to offer diagnostic genetic testing to patients with FALS and SALS (*p* = 1.2 × 10^−4^ and 2.6 × 10^−5^, respectively, Fisher exact test). Respondents' likelihood of recommending genetic testing was strongly influenced by the number and degree of relatives with ALS (figure 2C). When presented with a list of reasons for not offering testing for either SALS or FALS, the most common answer was the absence of any change in treatment plan (42.4%), then the cost of testing (37.9%), inadequate access to genetic counseling (25.8%), a belief that ALS genetics are not well enough understood in general (16.7%), or by the clinical team providing care (15.2%).

**Figure 3:**
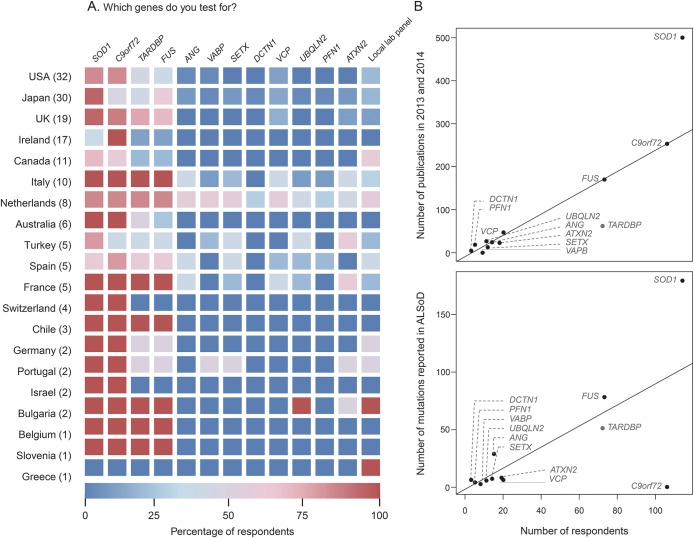
Choice of genes in genetic testing (A) Heat map of genes tested by respondents broken down by country. (B) The number of publications in amyotrophic lateral sclerosis (ALS) research concerning each gene as a function of the number of respondents who test for that gene (top) and the number of mutations reported in the ALS online database (ALSoD) for each gene as a function of the number of respondents who test for that gene (bottom).

Although there was some regional variation in the gene panel tested by respondents, there was a general consensus in the choice of testing for 4 main ALS genes (figure 3A): *SOD1* (68.3% of respondents test), *C9orf72* (63.5%), *TARDBP* (43.1%), and *FUS* (43.7%). Other known genes included in the survey received lower priority, and correlated with the number of publications that associate the gene with ALS (*SOD1* and *TARDBP* excluded; figure 3B; *r*^2^ = 0.98; *p* = 2 × 10^−8^) and the number of mutations that have been reported for the gene in the ALS online genetics database^16^ (*C9orf72* and *SOD1* excluded; figure 3B; *r*^2^ = 0.86; *p* = 1 × 10^−4^). Genetic testing for *C9orf72* was lower in Japan, but higher than expected given that this variant is rare in the Asian population (30% of Japanese respondents would test this gene vs 67% of non-Japanese respondents). Factors that influenced the selection of genes included the presence of a family history of FTD in addition to ALS (68.3%), the phenotype of the patient (59.3%), geography and ethnicity (21.6%), and in a minority, the advice of a specialist genetic counselor (12.0%).

### Personal preference and consistency.

**Figure 4:**
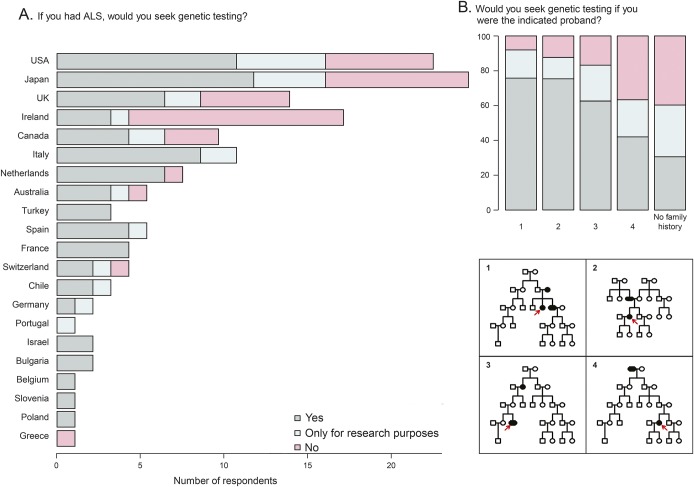
Number of respondents who would seek genetic testing if diagnosed with amyotrophic lateral sclerosis (ALS) (A) Would seek genetic testing (yes/no). (B) Would seek genetic testing if the indicated proband (yes/no).

The majority of respondents demonstrated consistency in their responses across various scenarios. Over half of those who responded stated that they would seek genetic testing if they had personally received a diagnosis of ALS (55.2%). While there was no significant difference between broad geographic regions, there was a considerable variation across different countries (*p* = 3.0 × 10^−3^, Fisher exact test). In some countries (for example, Ireland, United Kingdom, Japan, and United States), the majority of respondents stated that they would not seek testing or would test only for research purposes (figure 4A). Respondents' personal preferences for testing were also compared to answers they provided regarding their professional practice: 11.4% stated that they would offer testing to a patient with FALS (pedigree 1 in figures 2 and 4) but would only test themselves for research purposes or not at all, while 1.8% of respondents stated that they would test themselves but would not advise their patients to undergo testing. For SALS, these proportions were 28.7% and 8.2%, respectively.

### Presymptomatic testing.

**Figure 5:**
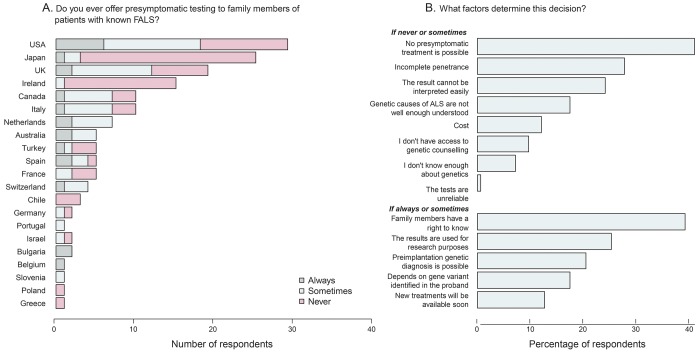
Respondents' opinions on presymptomatic testing (A) Would offer presymptomatic testing to family members of patients with known (FALS) (yes/no). (B) Factors that would determine the decision to offer presymptomatic testing to family members of patients with known familial amyotrophic lateral sclerosis (FALS).

Forty-eight percent of respondents never offer presymptomatic testing to family members of patients with FALS (figure 5A). These proportions differed by geographic region (*p* = 0.015, Fisher exact test), with respondents from Asia-Pacific stating never 1.8 times as frequently as those from the Americas (73.3% vs 40.5%) and 1.6 times as frequently as Europeans (47.2%). Specialists in ALS were significantly more likely to offer presymptomatic testing sometimes or always compared to nonspecialists (59.5% vs 14.8%, *p* = 2.83 × 10^−5^, Fisher exact test). Experienced ALS specialists (>15 years) were not significantly more likely than less experienced specialists to offer presymptomatic genetic testing to unaffected family members of patients with ALS (*p* = 1.0, Fisher exact test) but respondents who see more than 30 new patients with ALS per year were significantly more likely to offer presymptomatic testing (*p* = 1.7 × 10^−7^, Fisher exact test). Of the list of reasons presented to respondents for not testing, the most common selected was that no presymptomatic treatment is possible (51.9%, figure 5B), while the most common reason for offering presymptomatic testing was the belief that family members have a right to know (82.3%, figure 5B).

The answers of the respondents were used to compare whether they offer presymptomatic testing to patients and whether they would recommend testing to at-risk members of their own family. While most respondents were consistent, almost one-fifth of all respondents stated that they would offer presymptomatic testing to patients' families but would not recommend it to their own family, and 13% stated that they would recommend testing in their own family but would not recommend this to their patients.

## DISCUSSION

The true frequency of FALS has not been established, partly because the definition remains unclear. A meta-analysis of population-based registers undertaken prior to the discovery of the *C9orf72* repeat expansion suggested a FALS rate of 5.1%.^17^ However, a subsequent detailed family aggregation study, including cases identified through death certification and review of medical notes, suggests that 16% of patients have at least one other family member with ALS and 14% have a first-, second-, or third-degree relative with ALS.^18^ An audit of the Irish ALS register for patients diagnosed between 2013 and 2015 provides a rate of FALS of 16.61% for patients with 1 first- or 1 second-degree relative with ALS. This increases to 19.38% if first- or second-degree relatives with FTD are included, and is reduced to 3.81% if restricted only to those who have 2 or more first-degree relatives with ALS, demonstrating the range in the estimated rate of FALS, depending on the definition used, and the amount of information available (A. Vajda, unpublished data, 2016).

In the absence of a formal and evidence-based definition of FALS, it is difficult for clinicians to provide clear advice to patients and their families with ALS. This observation is reflected in our survey, in which 73.3% of respondents stated that there is no consensus as to the definition of FALS.

As would be expected, as the number of family members with ALS and their relatedness to the patient in question increased, so did the number of all respondents who accepted that definition of FALS. Compared to previous studies, a higher portion of respondents now consider the presence of a family history of FTD in the diagnosis of FALS, reflecting the growing recognition of the clinical overlap between the 2 disorders. Specialists and those who see more than 30 new patients with ALS per year were more inclusive in their diagnosis of FALS, with a higher likelihood of including those with a family history of FTD or those who appear sporadic by family history but test positive for a known ALS gene.

In terms of the use of diagnostic testing, most respondents offer genetic testing for 4 main genes (*SOD1*, *C9orf72*, *TARDBP*, and *FUS*) to those patients meeting their previously defined criterion for FALS, which is consistent with recommendations by the ITALSGEN consortium.^14^ Although we detected regional variation in the choice of genes, as would be expected given the importance of ancestral and geographic variation in gene frequencies, the rates of testing did not always reflect the prevalence of disease-causing variants. For example, 30% of respondents from Japan reported testing for *C9orf72* variants, despite this gene not contributing significantly to ALS in Japan,^19^ and 75% of Dutch respondents test for *SOD1*, which was not significantly different from the rate of testing in non-Dutch respondents (68%; *p* = 1.0, Fisher exact test), despite published evidence of low prevalence of *SOD1* disease-causing variants in the Netherlands.^9^

Our current knowledge of ALS genetics largely comes from the study of ancestral European (Europe, United States, Canada, and Australia) and East Asian populations.^14^ The frequency of known genes has not been established in African populations,^20^ and little is known about the population-based frequencies of ALS in the admixed populations of South America, the Middle East, or India. A number of genetically isolated populations with relatively homogenous genetic backgrounds have been reported, with strong evidence of founder effects in some regions. For example, more than 40% of Sardinian patients with ALS have a mutation in a known ALS gene (*TDP43* or *C9orf72*).^21^ Similarly, Northern Finland exhibits high rates of *C9orf72* repeat expansions and Northern Sweden has high rates of *SOD1* mutations.^22^ Recognition of the presence of a likely founder within a region could inform both genetic counseling and testing. However, it must also be noted that there is evidence of oligogenic inheritance in ALS, and that testing of a single gene variant in familial ALS could provide inaccurate information.^23^

We found that decisions surrounding genetic testing were less consistent in the case of apparently sporadic patients, with an almost even split between those respondents who reported that they sometimes offer and those who never offer diagnostic testing. Factors that determined the decision to offer testing included whether the neurologists were specialists in ALS and the number of patients seen per year, requests by patients, and recognition that some disease-associated variants are incompletely penetrant. How respondents address the implications of finding a pathogenic gene variant in non-FALS patients, and whether all respondents seek the advice and support of genetic counselors prior to diagnostic testing, was not addressed by our questionnaire. However, given that only 12% of all respondents reported that they select genes for testing following consultation with a counselor, it is likely that many ALS clinics do not have access to or utilize expert genetic counseling services, and it may also be that some patients are tested without full discussion with a qualified counselor as to the implications of a genetic diagnosis.

Another important factor in determining whether testing is offered may be regional variation in access to genetic testing through the health system and the reimbursement policies of insurance companies. The American ALS Association has estimated that the cost of testing for all known FALS genes is in the region of $6,000.^24^ While the implications of cost were not assessed by our study, there is considerable variation in the attitudes of insurance companies and health services with respect to funding the costs of counseling and testing. Counseling and testing may not be covered by some US-based insurance companies, while in regions with socialized health systems such as Italy, the process is funded if recommended by a specialist.^14^ This variation could have affected our findings.

Respondents were also divided regarding testing of presymptomatic family members of patients with FALS. Specialists in ALS differed significantly in their response from nonspecialists. In addition, a geographic variation was noted, with respondents from the United States and Europe being more likely to offer presymptomatic testing compared with their counterparts in Australia and Japan. The right to know was the commonest reason provided by respondents who offered presymptomatic testing, and a view that the implications of carrying a known variant are not known, and therefore cannot be reliably discussed with those wishing to undergo presymptomatic testing, was the commonest reason reported by respondents who do not routinely offer presymptomatic testing. An updated set of guidelines for providing presymptomatic genetic counseling and testing to people at high genetic risk for developing ALS will be of value to practitioners engaging with families with known ALS-causing variants.

A substantial number of respondents (44.8%) would not seek genetic testing if they had personally received a diagnosis of ALS. In general, respondents to this survey were consistent with respect to how they would approach testing of themselves and their family members, compared to what they recommend to their patients, although a minority were discordant. This discordance was not associated with geographic location, suggesting that it was driven by personal attributes of the clinicians rather than cultural determinants.

The complexity of ALS genetics renders the characterization of FALS problematic, and the provision of genetic counseling is limited by our current knowledge base. The pathogenicity of many reported gene variants in ALS remains unproven, and there is strong evidence to suggest that at least some are not disease-causing.^25^ The likely presence of oligogenic inheritance, genetic pleiotropy, and the absence of a clear phenotype/genotype correlation for many gene variants adds to the complexity. For example, *SOD1*, the first gene found to be associated with ALS, has over 160 reported mutations associated with phentotypes that range from a rapidly progressing form of the disease to a much milder, slowly progressing form.^26,27^ In the case of the *C9orf72* repeat expansion, healthy individuals typically have 10 or fewer repeats, and patients with ALS or FTD associated with this variant have between 30 and many thousands of repeats. However, an intermediate repeat number of 20–30 has been identified in both patients and controls, and further study is required to better understand the phenotype/genotype correlation for this mutation.^28,29^

Taken together, our data indicate an absence of consensus regarding the definition of FALS, and inconsistencies in the application of recent guidelines in the provision of genetic counseling and use of genetic testing in ALS. While this is not surprising given the complexity of ALS genetics, we detected significant differences in practice across geographic regions, and between specialists’ experience in managing many patients with ALS and those who follow relatively few patients, or do not have a special interest in the disease. It is likely that the observed variations in practice reflect not only differences in cultural attitudes to genetic testing, but also the level of expertise of the practitioner, and the availability of resources within the health care system of the country. Our data are limited by the study design. As the survey was disseminated by various networks, we cannot accurately calculate the response rate. Furthermore, the number of respondents (167) may be a limitation, given their spread over 21 countries. However, while the possibility of bias in response cannot be excluded, we have no reason to consider that the views of the respondents, many of whom described themselves as ALS specialists, were not representative.

These data suggest that the clinical application of genetic testing in symptomatic patients is not always evidence-based, and that genetic counseling of patients and their families does not occur routinely as a standard of care in all instances. Presymptomatic testing may sometimes occur with limited recognition of the presence of genetic pleiotropy and oligogenic inheritance. These findings suggest the need for evidence-based and consensus guidelines as to the most appropriate utilization of diagnostic and presymptomatic genetic testing in routine clinical management of patients with ALS and their extended families.

## Supplementary Material

Data Supplement

## GLOSSARY

ALS: amyotrophic lateral sclerosis
FALS: familial amyotrophic lateral sclerosis
FTD: frontotemporal dementia
SALS: sporadic amyotrophic lateral sclerosis
