# Supplementary material for: Genetic testing in ALS: A survey of current practices
Source: Neurology. 2017 Mar 7;88(10):991–9. doi: 10.1212/WNL.0000000000003686 (PMC5333513; doi:10.1212/WNL.0000000000003686)
Supplement: Data Supplement [file supp_88_10_991__index.html]

Genetic testing in ALS — Data Supplement 

# Genetic testing in ALS

## Data Supplement

**Neurology® data supplements are not copyedited before publication. Published editorials and translations have been copyedited.  
 © 2017 American Academy of Neurology.  
  
 Files in this Data Supplement:**

- Survey - PDF
