# Supplementary material for: Genetic testing in ALS: A survey of current practices
Source: Neurology. 2017 Mar 7;88(10):991–9. doi: 10.1212/WNL.0000000000003686 (PMC5333513; doi:10.1212/WNL.0000000000003686)
Supplement: Data Supplement [file supp_WNL.0000000000003686_Survey.pdf]

Thank you for taking ten minutes of your time to answer some questions about your clinical views on diagnostic genetic testing in Familial Amyotrophic Lateral Sclerosis (FALS). There are no correct answers. All questionnaires are confidential and you will not be asked your name.

Your views, as a researcher and clinician, are much valued.

Kind regards,

Owen Thorpe and Alice Vajda (Trinity College Dublin)

Supported by

Sharon Abrahams, Ammar Al-Chalabi, Susan Byrne and Orla Hardiman

***Sex:**

***Country in which you practice:**

***City in which you practice:**

***Do you work in an:**

***What is your clinical role?**

***How many new Amyotrophic Lateral Sclerosis (ALS) patients do you see each year?**

***Do you have a special interest in ALS?**

If yes, for how many years have you been specialising in the field of ALS?

Q1. Do you think there is a standard definition among neurologists for Familial ALS (FALS)?

- ◯ Yes
- ◯ No

Q2. In your clinical practice, which of the following statements fits your definition of FALS? (*Please tick as many as needed*):

- ☐ A patient with ALS who has **one first** degree relative affected with ALS
- ☐ A patient with ALS with one first degree relative with Frontotemporal Dementia (FTD)
- ☐ A patient with ALS who has **two or more first** degree relatives affected with ALS
- ☐ A person with ALS who has two or more first degree relatives with FTD
- ☐ A patient with ALS with either **one first or one second** degree relative with ALS
- ☐ A patient with ALS with either one first or one second degree relative with FTD
- ☐ A patient with ALS with **any relative** with ALS no matter how distant
- ☐ A patient with sporadic ALS who tests positive for a known ALS gene (e.g. C9orf72, SOD1, FUS, TARDBP etc)
- ☐ A patient with ALS who has mentioned they have or had a distant affected relative with a condition that could be ALS

If none of the above definitions match the one you use, please feel free to describe your definition in the text box below.

Note: 1st degree relative: Parent, Sibling, Child

2nd degree relative: Grandparent, Aunt/Uncle, Niece/Nephew

Q3. Do you offer diagnostic genetic testing for patients with a family history of ALS? (as you have defined above)

- ○ Yes/Sometimes
- ○ No

Q4. Do you offer genetic testing for patients with no known family history of ALS?

- ○ Yes/Sometimes
- ○ No

Q5. Which genes do you test for? (*Please tick as many as needed*):

- ☐ N/A as I never test.
- ☐ SOD1
- ☐ C9orf72
- ☐ TARDBP
- ☐ FUS
- ☐ ANG
- ☐ VABP
- ☐ SETX
- ☐ DCTN1
- ☐ VCP
- ☐ UBQLN2
- ☐ PFN1
- ☐ ATXN2
- ☐ Don’t know
- ☐ Depends on the phenotype
- ☐ ALS gene panel designated by local laboratory
- ☐ Other (please specify)

Q6. When you decide to test, what factors determine your selection of genes? (*Please tick as many as needed*):

- ☐ Phenotype of patient
- ☐ The patient has a family history of FTD in addition to ALS
- ☐ Geography and ethnicity (rate of a gene in your particular country or in particular ethnic group)
- ☐ Genetic counsellors’ advice
- ☐ ALS gene panel designated by local laboratory

Q7. If you decide to carry out genetic testing on affected individuals, what factors determine this decision? (*Please tick as many as needed*):

- [ ] I always test if the patient has FALS and has not been previously tested
- [ ] I test if the patient requests genetic testing (regardless of whether the disease is familial)
- [ ] I only test if the patient has FALS and they request testing
- [ ] I test for research purposes and do not disclose the result to the patient or family
- [ ] I never test

Q8. If you do not routinely carry out diagnostic genetic testing on affected individuals with familial ALS, what factors determine this decision? (Please tick as many as needed):

- [ ] N/A as I routinely test in cases of FALS
- [ ] Inadequate access to genetic counselling
- [ ] No change in treatment plan would occur
- [ ] Cost
- [ ] The genetic causes of ALS are not well enough understood
- [ ] I don't know enough about genetics
- [ ] The tests are unreliable

Other (please specify)

Q9. Do you ever offer pre-symptomatic testing to family members of patients with known FALS?

- ○ Never
- ○ Sometimes
- ○ Always

Q10. If never or sometimes, what factors determine this decision? (*Please tick as many as needed):*

- ☐ I don’t have access to genetic counselling
- ☐ No pre-symptomatic treatment is possible
- ☐ Cost
- ☐ The genetic causes of ALS are not well enough understood
- ☐ I don’t know enough about genetics
- ☐ The tests are unreliable
- ☐ The result cannot be interpreted easily
- ☐ Incomplete penetrance

Other (please specify)

Q11. If always or sometimes, what factors determine this decision? (Please tick as many as needed):

- ☐ Family members have a right to know
- ☐ New treatments will be available soon
- ☐ The results are used for research purposes
- ☐ Pre-implantation genetic diagnosis is possible
- ☐ Depends on which gene variant has been identified in the proband.

Q12. If YOU were a member of an ALS kindred in which your mother and brother had died of a disease resembling ALS would you recommend genetic testing for your affected brother?

- ○ Yes
- ○ No

If yes, which genes would you test for? (*Please tick as many as needed*):

- ☐ N/A as I wouldn't test.
- ☐ SOD1
- ☐ C9orf72
- ☐ TARDBP
- ☐ FUS
- ☐ ANG
- ☐ VABP
- ☐ SETX
- ☐ DCTN1
- ☐ VCP
- ☐ UBQLN2
- ☐ PFN1
- ☐ ATXN2
- ☐ Don’t know
- ☐ Depends on the phenotype
- ☐ ALS gene panel designated by local laboratory
- ☐ Other (please specify)

Q13. If YOU were a <u>member of an ALS kindred</u> with a known pathogenic gene variant, would you recommend pre-symptomatic genetic testing for at risk members of your family?

- ◯ Yes
- ◯ No
- ◯ Only for first degree relatives
- ◯ Only for those planning a family
- ◯ Would depend on the gene variant

Other (please specify)

If yes, which genes would you test for? (*Please tick as many as needed*):

- [ ] N/A as I wouldn't test.
- [ ] SOD1
- [ ] C9orf72
- [ ] TARDBP
- [ ] FUS
- [ ] ANG
- [ ] VABP
- [ ] SETX
- [ ] DCTN1
- [ ] VCP
- [ ] UBQLN2
- [ ] PFN1
- [ ] ATXN2
- [ ] Don’t know
- [ ] Depends on the phenotype
- [ ] ALS gene panel designated by local laboratory
- [ ] Other (please specify)

Q14. If YOU were a member of a kindred in which one of your brothers and one of your aunts had ALS (or ALSFTD), but you were asymptomatic, would YOU undergo PRESYMPTOMATIC genetic testing <u>if your affected family members had refused to be tested?</u>

If yes, which genes would you test for? (*Please tick as many as needed*)

- [ ] N/A as I wouldn't test
- [ ] SOD1
- [ ] C9orf72
- [ ] TARDBP
- [ ] FUS
- [ ] ANG
- [ ] VABP
- [ ] SETX
- [ ] DCTN1
- [ ] VCP
- [ ] UBQLN2
- [ ] PFN1
- [ ] ATXN2
- [ ] Don’t know
- [ ] Depends on the phenotype
- [ ] ALS gene panel designated by local laboratory
- [ ] Other (please specify)

Q15. If this patient presented to your clinic with the following family history of ALS, would you recommend genetic testing?

Filled in squares/circles represent a person who has ALS in the patient’s family pedigree

Your patient is annotated by the red arrow

A.

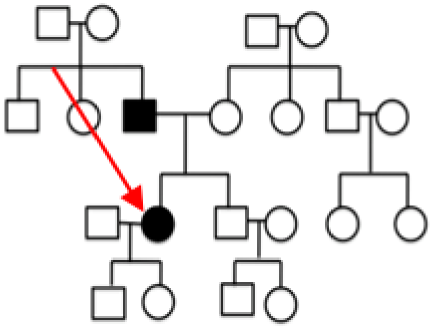

If yes, which genes would you test for? (*Please tick as many as needed*)

- [ ] N/A as I wouldn't test
- [ ] SOD1
- [ ] C9orf72
- [ ] TARDBP
- [ ] FUS
- [ ] ANG
- [ ] VABP
- [ ] SETX
- [ ] DCTN1
- [ ] VCP
- [ ] UBQLN2
- [ ] PFN1
- [ ] ATXN2
- [ ] Don’t know
- [ ] Depends on the phenotype
- [ ] ALS gene panel designated by local laboratory
- [ ] Other (please specify)

B.

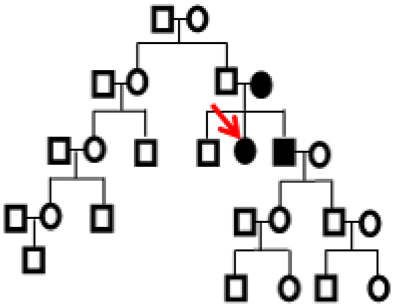

If yes, which genes would you test for? (*Please tick as many as needed*)

- [ ] N/A as I wouldn't test
- [ ] SOD1
- [ ] C9orf72
- [ ] TARDBP
- [ ] FUS
- [ ] ANG
- [ ] VABP
- [ ] SETX
- [ ] DCTN1
- [ ] VCP
- [ ] UBQLN2
- [ ] PFN1
- [ ] ATXN2
- [ ] Don’t know
- [ ] Depends on the phenotype
- [ ] ALS gene panel designated by local laboratory
- [ ] Other (please specify)

c.

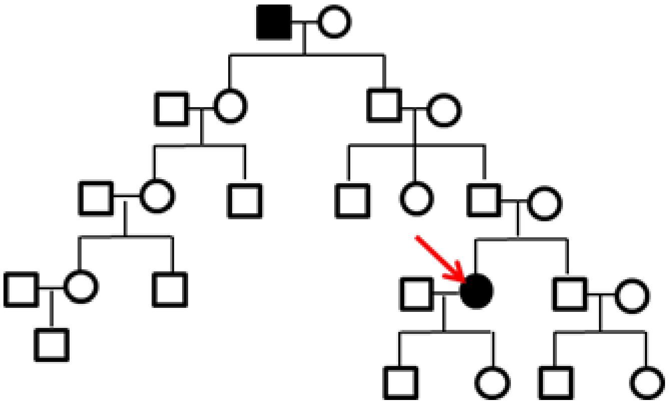

If yes, which genes would you test for? (*Please tick as many as needed*)

- ☐ N/A as I wouldn't test
- ☐ SOD1
- ☐ C9orf72
- ☐ TARDBP
- ☐ FUS
- ☐ ANG
- ☐ VABP
- ☐ SETX
- ☐ DCTN1
- ☐ VCP
- ☐ UBQLN2
- ☐ PFN1
- ☐ ATXN2
- ☐ Don't know
- ☐ Depends on the phenotype
- ☐ ALS gene panel designated by local laboratory
- ☐ Other (please specify)

D.

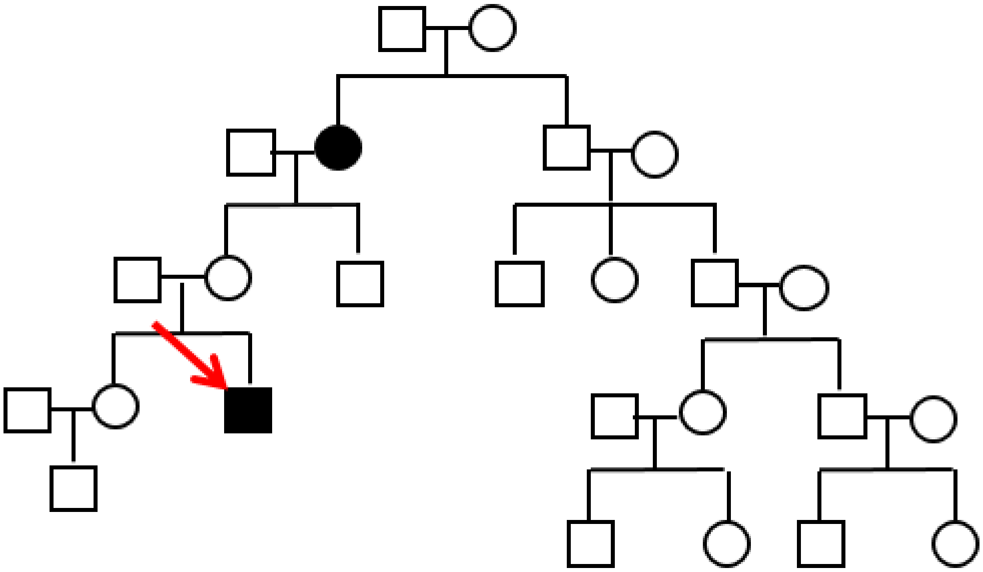

If yes, which genes would you test for? (*Please tick as many as needed*)

- [ ] N/A as I wouldn't test
- [ ] SOD1
- [ ] C9orf72
- [ ] TARDBP
- [ ] FUS
- [ ] ANG
- [ ] VABP
- [ ] SETX
- [ ] DCTN1
- [ ] VCP
- [ ] UBQLN2
- [ ] PFN1
- [ ] ATXN2
- [ ] Don't know
- [ ] Depends on the phenotype
- [ ] ALS gene panel designated by local laboratory
- [ ] Other (please specify)

Q16. If this patient with ALS presented to your clinic, and their mother and sister had a <u>history of FTD but not ALS</u> would you offer genetic testing?

Filled in black squares/circles represent a person who has ALS or FTD in the patient's family pedigree. The patient's mother and sister had a history of FTD. Your patient has ALS and is annotated with the red arrow.

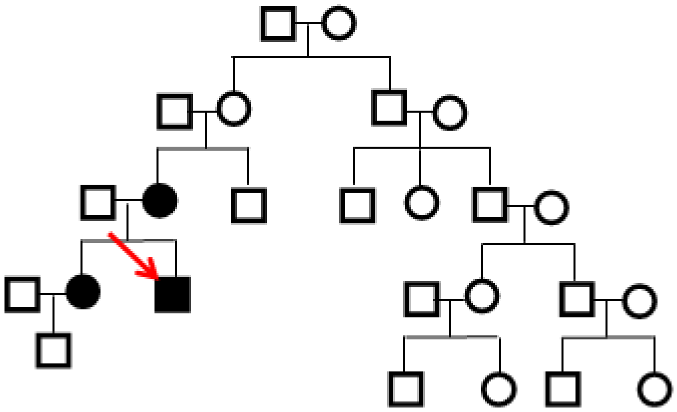

If yes, which genes would you test for? (*Please tick as many as needed*)

- ☐ N/A as I wouldn't test
- ☐ SOD1
- ☐ C9orf72
- ☐ TARDBP
- ☐ FUS
- ☐ ANG
- ☐ VABP
- ☐ SETX
- ☐ DCTN1
- ☐ VCP
- ☐ UBQLN2
- ☐ PFN1
- ☐ ATXN2
- ☐ Don't know
- ☐ Depends on the phenotype
- ☐ ALS gene panel designated by local laboratory
- ☐ Other (please specify)

Q17. IF YOU YOURSELF HAD ALS, would you seek genetic testing?

If yes, would you seek genetic testing:

A. If you had no family history of ALS?

B. If you were this person?

Filled in squares/circles represent a person who has ALS in your family pedigree

You are annotated by the red arrow

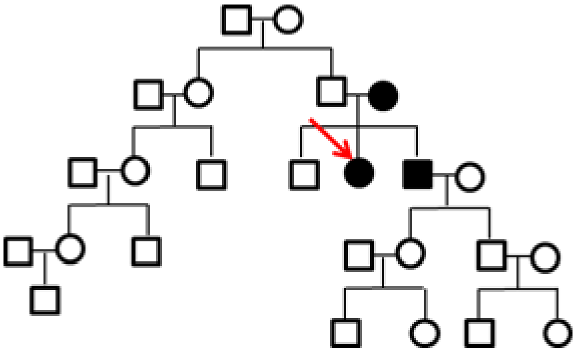

If yes, which genes would you test for? (*Please tick as many as needed*)

- [ ] N/A as I wouldn't seek testing
- [ ] SOD1
- [ ] C9orf72
- [ ] TARDBP
- [ ] FUS
- [ ] ANG
- [ ] VABP
- [ ] SETX
- [ ] DCTN1
- [ ] VCP
- [ ] UBQLN2
- [ ] PFN1
- [ ] ATXN2
- [ ] Don't know
- [ ] Depends on the phenotype
- [ ] ALS gene panel designated by local laboratory
- [ ] Other (please specify)

C. If you were this person?

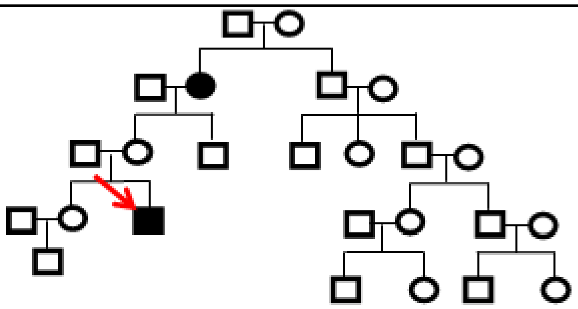

If yes, which genes would you test for? (*Please tick as many as needed*)

- ☐ N/A as I wouldn't seek testing
- ☐ SOD1
- ☐ C9orf72
- ☐ TARDBP
- ☐ FUS
- ☐ ANG
- ☐ VABP
- ☐ SETX
- ☐ DCTN1
- ☐ VCP
- ☐ UBQLN2
- ☐ PFN1
- ☐ ATXN2
- ☐ Don't know
- ☐ Depends on the phenotype
- ☐ ALS gene panel designated by local laboratory
- ☐ Other (please specify)

D. If you were this person?

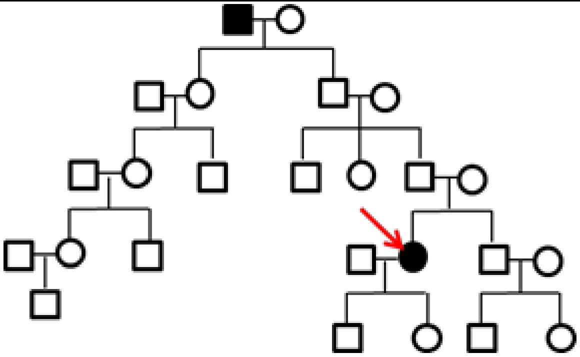

If yes, which genes would you test for? (*Please tick as many as needed*)

- ☐ N/A as I wouldn't seek testing
- ☐ SOD1
- ☐ C9orf72
- ☐ TARDBP
- ☐ FUS
- ☐ ANG
- ☐ VABP
- ☐ SETX
- ☐ DCTN1
- ☐ VCP
- ☐ UBQLN2
- ☐ PFN1
- ☐ ATXN2
- ☐ Don’t know
- ☐ Depends on the phenotype
- ☐ ALS gene panel designated by local laboratory
- ☐ Other (please specify)

E. If you were this person?

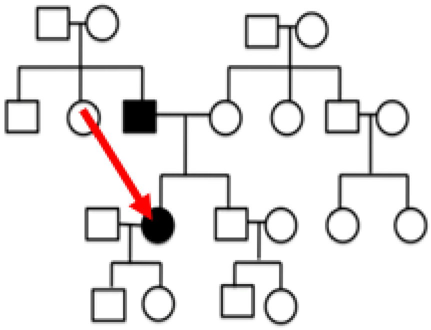

If yes, which genes would you test for? (*Please tick as many as needed*)

- ☐ N/A as I wouldn't seek testing
- ☐ SOD1
- ☐ C9orf72
- ☐ TARDBP
- ☐ FUS
- ☐ ANG
- ☐ VABP
- ☐ SETX
- ☐ DCTN1
- ☐ VCP
- ☐ UBQLN2
- ☐ PFN1
- ☐ ATXN2
- ☐ Don't know
- ☐ Depends on the phenotype
- ☐ ALS gene panel designated by local laboratory
- ☐ Other (please specify)

Q18. If this <u>UNAFFECTED FIRST DEGREE RELATIVE</u> sought pre-symptomatic testing for diagnostic purposes, and the gene variant in the family were <u>known</u>, would you advise in favour of testing?

Filled in squares/circles represent a person who has ALS in the patient's family pedigree

Your patient is annotated by the red arrow

A.

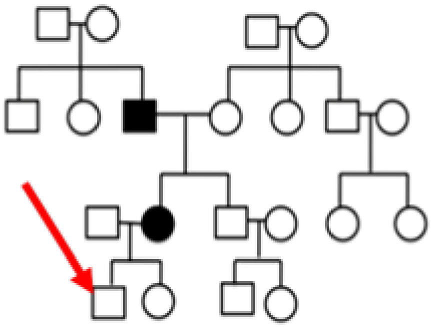

Please explain your answer in the text box below:

B.

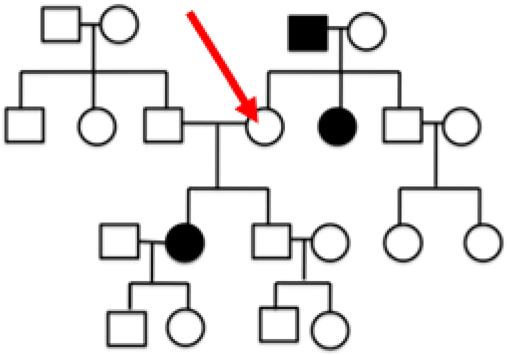

Please explain your answer in the text box below:

C.

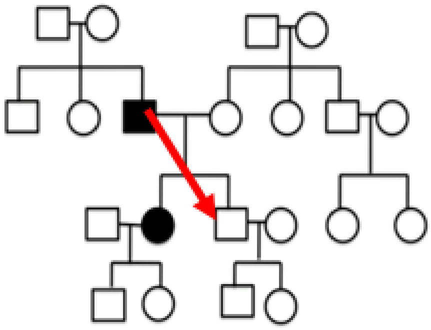

Please explain your answer in the text box below:

D.

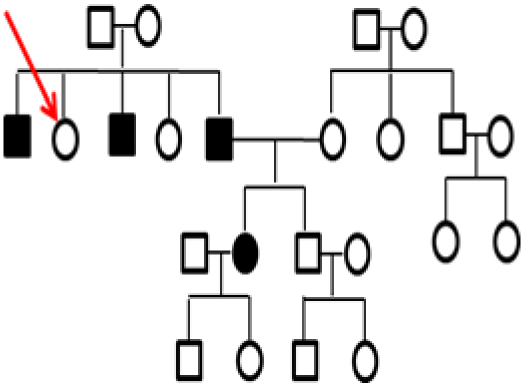

Please explain your answer in the text box below:

Q19. If this UNAFFECTED FIRST DEGREE RELATIVE sought pre-symptomatic testing for diagnostic purposes, would you advise in favour if the gene variant had not been established within the kindred?

Filled in squares/circles represent a person who has ALS in the patient's family pedigree

Your patient is annotated by the red arrow

A.

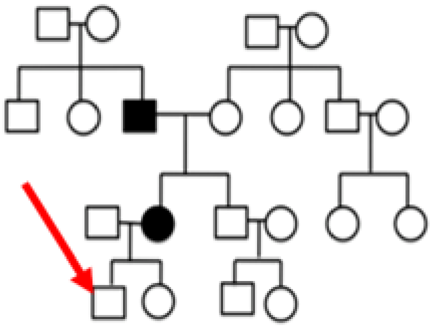

Please explain your answer in the text below below:

B.

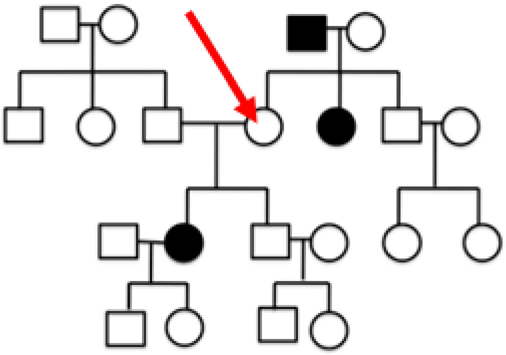

Please explain your answer in the text below below:

C.

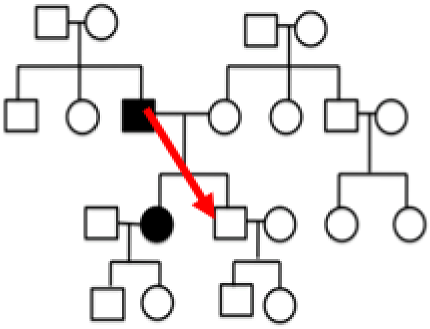

Please explain your answer in the text below below:

D.

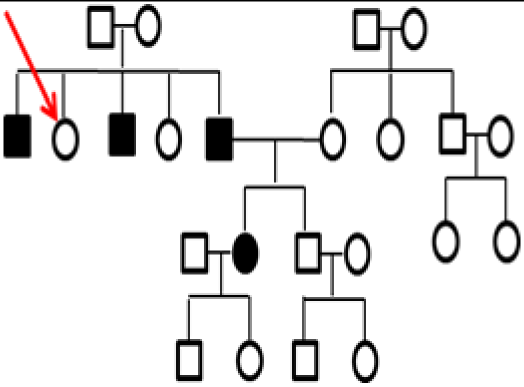

Please explain your answer in the text below below:

The following questions will ask you about your personal views, rather than professional ones, towards diagnostic genetic testing in ALS.

Q20. If <u>YOU were this unaffected person in this kindred</u> , would you seek genetic testing for diagnostic purposes, assuming the variant in the family is <u>known</u>?

Filled in squares/circles represent a person who has ALS in your family pedigree

You are annotated by the red arrow

A.

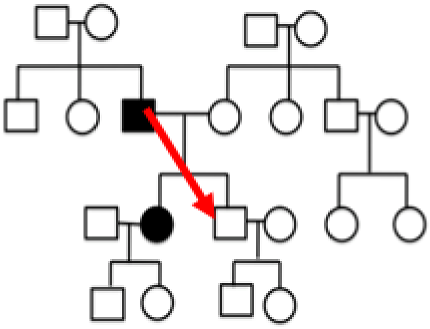

If no, what would influence your decision not to undergo genetic testing? (Please tick as many as needed):

- [ ] I don't have access to genetic counselling
- [ ] No pre-symptomatic treatments is possible
- [ ] Cost
- [ ] The genetic causes of ALS are not well enough understood
- [ ] I don't know enough about ALS genetics
- [ ] The tests are unreliable
- [ ] The results cannot be interpreted easily
- [ ] Incomplete penetrance

Other (please specify)

B.

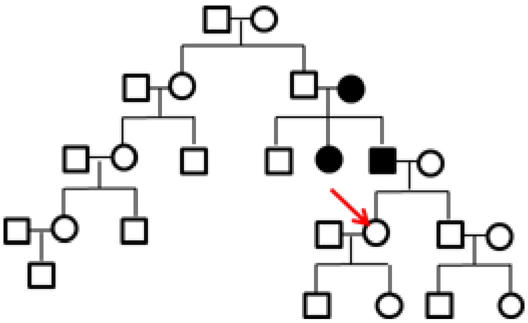

If no, what would influence your decision not to undergo genetic testing? (Please tick as many as needed):

- [ ] I don't have access to genetic counselling
- [ ] No pre-symptomatic treatments is possible
- [ ] Cost
- [ ] The genetic causes of ALS are not well enough understood
- [ ] I don't know enough about ALS genetics
- [ ] The tests are unreliable
- [ ] The results cannot be interpreted easily
- [ ] Incomplete penetrance

Other (please specify)

c.

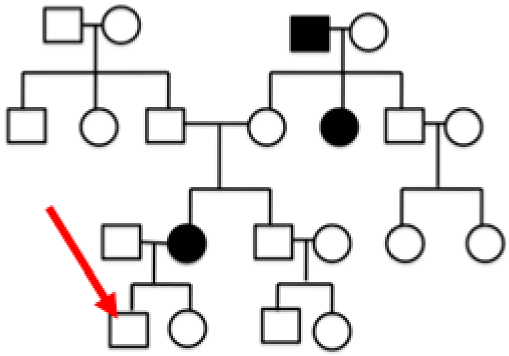

If no, what would influence your decision not to undergo genetic testing? (Please tick as many as needed):

- [ ] I don't have access to genetic counselling
- [ ] No pre-symptomatic treatments is possible
- [ ] Cost
- [ ] The genetic causes of ALS are not well enough understood
- [ ] I don't know enough about ALS genetics
- [ ] The tests are unreliable
- [ ] The results cannot be interpreted easily
- [ ] Incomplete penetrance

Other (please specify)

Q21. If YOU were this person in this kindred, would you seek testing for diagnostic purposes if the genetic status of your affected sister, father and daughter were unknown?

Filled in squares/circles represent a person who has ALS in your family pedigree

You are annotated by the red arrow

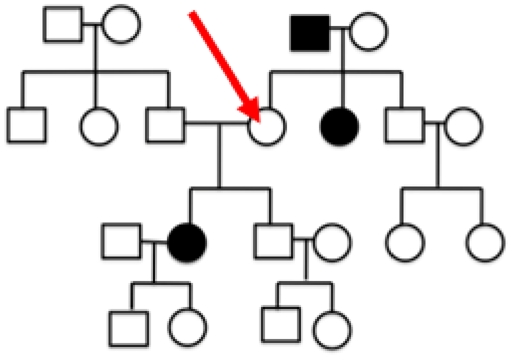

Briefly, please explain your answer

If you chose yes, which genes would you test for? (*Please tick as many as needed*):

- ☐ N/A as I wouldn't seek testing
- ☐ SOD1
- ☐ C9orf72
- ☐ TARDBP
- ☐ FUS
- ☐ ANG
- ☐ VABP
- ☐ SETX
- ☐ DCTN1
- ☐ VCP
- ☐ UBQLN2
- ☐ PFN1
- ☐ ATXN2
- ☐ Don't know
- ☐ Depends on the phenotype
- ☐ ALS gene panel designated by local laboratory
- ☐ Other (please specify)

We would like to thank you for your time and effort in completing this survey. Your views and opinions are greatly appreciated. If there is anything you would like to add, please feel free to do so in the text box below.

Comment:
